# Magnetic Biochar Obtained by Chemical Coprecipitation and Pyrolysis of Corn Cob Residues: Characterization and Methylene Blue Adsorption

**DOI:** 10.3390/ma16083127

**Published:** 2023-04-15

**Authors:** Norma Araceli Guel-Nájar, Jorge Carlos Rios-Hurtado, Elia Martha Muzquiz-Ramos, Gloria I. Dávila-Pulido, Adrián A. González-Ibarra, Aurora M. Pat-Espadas

**Affiliations:** 1 Facultad de Metalurgia, Universidad Autónoma de Coahuila, Carretera 57 Km 5, Monclova 25710, Coahuila, Mexico; normaguel@uadec.edu.mx; 2 Facultad de Ciencias Químicas, Universidad Autónoma de Coahuila, Blvd. Venustiano Carranza S/N, República, Saltillo 25280, Coahuila, Mexico; 3 Escuela Superior de Ingeniería, Universidad Autónoma de Coahuila, Boulevard Adolfo López Mateos S/N, Independencia, Nueva Rosita 26830, Coahuila, Mexico; gloriadavila@uadec.edu.mx (G.I.D.-P.); gonzalez-adrian@uadec.edu.mx (A.A.G.-I.); 4 CONACyT, Estación Regional del Noroeste del Instituto de Geología de la UNAM, Luis D Colosio S/N Esquina Madrid, Hermosillo 83200, Sonora, Mexico; apespadas@geologia.unam.mx

**Keywords:** biochar, corn cob, magnetic composite, coprecipitation, methylene blue

## Abstract

Biochar is a carbonaceous and porous material with limited adsorption capacity, which increases by modifying its surface. Many of the biochars modified with magnetic nanoparticles reported previously were obtained in two steps: first, the biomass was pyrolyzed, and then the modification was performed. In this research, a biochar with Fe_3_O_4_ particles was obtained during the pyrolysis process. Corn cob residues were used to obtain the biochar (i.e., BCM) and the magnetic one (i.e., BCM_Fe_). The BCM_Fe_ biochar was synthesized by a chemical coprecipitation technique prior to the pyrolysis process. The biochars obtained were characterized to determine their physicochemical, surface, and structural properties. The characterization revealed a porous surface with a 1013.52 m^2^/g area for BCM and 903.67 m^2^/g for BCM_Fe_. The pores were uniformly distributed, as observed in SEM images. BCM_Fe_ showed Fe_3_O_4_ particles on the surface with a spherical shape and a uniform distribution. According to FTIR analysis, the functional groups formed on the surface were aliphatic and carbonyl functional groups. Ash content in the biochar was 4.0% in BCM and 8.0% in BCM_Fe_; the difference corresponded to the presence of inorganic elements. The TGA showed that BCM lost 93.8 wt% while BCM_Fe_ was more thermally stable due to the inorganic species on the biochar surface, with a weight loss of 78.6%. Both biochars were tested as adsorbent materials for methylene blue. BCM and BCM_Fe_ obtained a maximum adsorption capacity (q_m_) of 23.17 mg/g and 39.66 mg/g, respectively. The obtained biochars are promising materials for the efficient removal of organic pollutants.

## 1. Introduction

Due to rapid population growth, accelerated urbanization, and industrialization, the volume and diversity of agro-industrial wastes have increased. These wastes are considered a significant source of pollution due to poor management and improper disposal [1,2] (Awogbemi and Kallon, 2022; Karić et al., 2022). A strategy to reduce the disposed residues is to prepare new materials for different applications. Biochar is a solid material obtained from the thermochemical conversion of biomass in an oxygen-limited environment [3]. It is a carbon-rich organic material that is prepared by heating biomass. Depending on the feedstock used to obtain it, the properties of the biochar such as its high specific surface area, microporosity, and ion exchange capacity can be improved [4,5]. Furthermore, corn residues are suitable to obtain biochar due to their lignocellulosic biomass, which favors its formation [6,7].

The use of biochar as an adsorbent material has been widely studied; however, the adsorption efficiency can be improved. Surface activation or modification with chemical reagents, steam and gas, mineral oxides, organic compounds, clay minerals, and microorganisms has been widely reported [8,9,10]. Composite biochar is prepared by impregnating the surface with metal oxides to modify the surface properties and increase the adsorption capacity [10,11].

One of the ideal methods of surface modification is with magnetite (i.e., Fe_3_O_4_). This oxide is non-toxic, can improve the adsorption capacity towards various pollutants, and provides strong magnetic properties. Ion exchange, complexation, electrostatic attraction, and metal–π interaction are mechanisms that make adsorption techniques suitable. Due to its magnetic properties, Fe_3_O_4_ can be used to recycle biochar, as iron metal oxides allow adsorbent recovery [12,13,14]. This makes it important to obtain magnetic biochars for the removal of water pollutants. Santhosh et al. synthesized a magnetic biochar from sewage sludge and woodchips, for Cr (VI) removal [15]. A magnetic biochar in a single-step method was obtained for the removal of organic pollutants by Chen et al. (2022) [16]. A filamentous fungus, *Trichoderma reesei,* was used as a template for 3D biochar obtention. 

Water pollution is one of the challenges facing society [17]. One of the main sources of pollutants are organic dyes such as methylene blue (MB). This dye is widely used in the textile, food, cosmetic, and pharmaceutical industries; it usually causes negative effects on photosynthetic activity [17,18,19]. For this reason, it is important to remove dyes using adsorbent materials, such as biochars.

To increase the MB adsorption capacity, a wide variety of precursors have been used to obtain biochars with modified surfaces. Mu et al. (2022) obtained a magnetic biochar by slow pyrolysis of tea residue powder with FeCl_3_ solution impregnation. The authors studied MB adsorption as a dye model and described the adsorption process due to the main interactions of pore filling, hydrogen bonding, π–π interactions, and electrostatic attraction [20]. Güleç et al. (2022) prepared biochars from seaweed and rapeseed, obtaining a q_m_ of 117.6 and 70.9 mg/g, respectively [21]. Zeng et al. (2021) prepared a sludge-based magnetic biochar by pyrolysis and studied MB adsorption [22]. Li et al. (2022) made a biochar from moringa seed shells. In the study, Fe_3_O_4_ was added to activate the surface of the biochar, and a high removal of MB was observed [23]. 

Therefore, in this work, biochars were obtained from corn cob residues by pyrolysis. A pure biochar and a magnetite impregnated with magnetite by the coprecipitation technique prior to the pyrolysis process were obtained. Both biochars were characterized to determine their physicochemical, surface, and structural properties. MB adsorption was tested to understand the behavior of biochars when modified with Fe_3_O_4_.

## 2. Materials and Methods

### 2.1. Reagents

To obtain biochar, corn cob waste from the municipality of Frontera, Coahuila, was used as a precursor. The chemicals used are of reagent grade: hydrochloric acid (CTR Scientific, Monterrey, MX), ferric chloride hexahydrate (FeCl_3_·6H_2_O), ferrous chloride tetrahydrate (FeCl_2_·4H_2_O), and sodium hydroxide (Fermont, Monterrey, MX).

### 2.2. Experimental Procedures

#### 2.2.1. Pretreatment of Samples

The corn cob waste was cut into small pieces of approximately 3 cm in length, then washed with distilled water. A part of the cleaned waste was dried at 100 °C in an ECOSHEL 9053A oven (LABOTECA, Monterrey, MX) for 24 h. The remaining corn cob was passed through the chemical coprecipitation process.

#### 2.2.2. Chemical Coprecipitation of Magnetite in Corn Cob Waste

The coprecipitation method was based on the work of Rodriguez et al. (2019) [24]. A solution was prepared in a beaker with 50 mL of deionized water, FeCl_3_·6H_2_O and FeCl_2_·4H_2_O at a 2:1 ratio according to Equation (1):

(1) $$2{\mathrm{Fe}}^{3+}+{\mathrm{Fe}}^{2+}+8{\mathrm{OH}}^{-}\to {\mathrm{Fe}}_{3}O_{4}+4H_{2}O$$

The solution was heated at 50 °C for 15 min. Corn cobs cleaned of waste were placed in contact with 5 mL of Fe^3+^:Fe^2+^ ion solution. Then, 2 mL of concentrated NaOH was added by dripping and subsequently dried at 100 °C for 24 h.

#### 2.2.3. Obtaining Biochar from Corn Cob by Pyrolysis

The research of Lee et al. (2017) and Xie et al. (2021) was used to develop corn cob pyrolysis [25,26]. Dried corn cob residues were placed in a CARBOLITE™ CTF horizontal tube furnace (Fischer Scientific, Monterrey, MX) and heated to 500 °C for 1 h. The heating rate and N_2_ flow rate were varied. The obtained sample was allowed to cool slowly at room temperature. Biochar was labeled as BCM, and the modified biochar as BCM_Fe_.

#### 2.2.4. Characterization of Biochar

The biochar yield was determined according to the procedure reported by Qin et al. (2020), as well as the ash content using ASTM D2866-94 (ASTM, n.d.) [27,28]. In addition, SEM and EDS analysis were performed on a HITACHI model SU8230 cold cathode SEM machine (Ciudad de México, MX) with backscattered electron and secondary electron detectors; FTIR analysis with a PerkinElmer FTIR Spectrometer Frontier (Monterrey, Mexico) in a range of 4000 to 600 cm^−1^ using 32 scans and 4 cm^−1^ resolution; XRD on a Bruker D8 ADVANCE model (Ciudad de México, MX) (CuKa: 1.54 Å, 40 mA and 40 kV); XRF analysis on a Malvern Panalytical Epsilon 1 model (Monterrey, MX); and TGA on a TA Instruments TGA 550 (Ciudad de México, MX), in a temperature range of 30 °C to 700 °C at a heating rate of 10 °C/min, using air atmosphere; and N_2_ physisorption at 77 K was performed in a BEL BELSORP-miniX surface analyzer (Ciudad de México, MX).

#### 2.2.5. Methylene Blue Adsorption Efficiency and Kinetic Models

MB adsorption efficiency was determined for both biochars. In 15 mL conical tubes, 0.02 g of biochar and 10 mL of an MB solution at different concentrations (i.e., 1, 2, 4, 8, 10, and 25 ppm) were placed in contact. The pH was adjusted to 7 ± 0.2 in an Orion StarA2110 potentiometer (Monterrey, MX). The initial concentration (C_o_) absorbance of each of the solutions was measured in a Mettler Toledo UV-Vis instrument model UV5 (Ciudad de México, MX) (λ = 664 nm). Then, the tubes were placed in a LabTech LSI-3016A incubator (El Crisol, Monterrey, México) at 25 °C and 150 rpm for 24 h. Once equilibrium was reached, the remaining concentration (C_e_) values were measured by UV-Vis. The maximum adsorption capacity was calculated according to Equation (2) and fitted to the Langmuir isotherm model according to Equation (3):

(2) $$\mathrm{Qe}=\frac{\left(C_{O}-C_{e}\right)\left(V\right)}{m\;}$$

(3) $$q_{e}=\frac{q_{\max}k_{L}C_{e}}{1+k_{L}C_{e}}$$

where:

$q_{e}$ = adsorption capacity at equilibrium, mg·g^−1^

$C_{o}$ = initial concentration of MB solution, mg·L^−1^

$C_{e}$ = final concentration of MB solution, mg·L^−1^

V = solution volume, L

m = adsorbent mass, g

$q_{\max}$ = maximum adsorption capacity, mg·g^−1^

$k_{L}$ = adsorption energy constant, L·mg^−1^

For kinetic experiments, 60 mL of a 10 ppm MB solution and 60 mg of the material were placed in a beaker. Sampling was carried out during 0, 1, 3, 3, 5, 10, 20, 40, and 60 min. The concentration was measured by a UV/Vis spectrometer. The data obtained were adjusted to the pseudo-second-order model using the following equation (Equation (4)):

(4) $$\frac{t}{q_{t}}=\frac{1}{k_{2}{\left(q_{e}\right)}^{2}}+\frac{1}{q_{e}}t$$

where: 

*t* = time, min

*q_t_* = adsorption capacity at time, mg·g^−1^

*q_e_* = adsorption capacity at equilibrium, mg·g^−1^

*k*_2_ = PSO constant (g·mg^−1^·g^−1^)

## 3. Results and Discussions

### 3.1. Biochar Yield and Ash Percentage

It is important to dry the corn cob samples prior to pyrolysis in order to calculate the amount of biochar obtained. Besides, subjecting wet feedstock samples to pyrolysis would consume energy, and carbonization would last longer [8]. In addition, a large amount of moisture in the biomass leads to the production of liquid by-products [29]. The pyrolysis experimental conditions of the BCM samples and the yield are shown in Table 1. The N_2_ flow rate was tested, and for a 50 mL/min rate, the yield was 5.60%, and for a 35 L/min rate, the yield was 28.48%. Moreover, different heating ramps were tested. The results showed that when a lower heating ramp was used (5 °C/min), the biochar yield was 26.91%. However, a higher heating ramp (10 °C/min) produced a biochar yield of 28.48% and reduced the time needed to reach the pyrolysis temperature. In addition, raw and cooked corn cob (180 °C, 1 h) conditions were tested, and no significant difference in biochar yield was observed. Therefore, to obtain a higher biochar yield, an N_2_ flow rate of 35 mL/min and a heating ramp of 10 °C/min were used, in which an average yield of 28.48 ± 0.098% was obtained.

In Table 2, the ash contents of different samples are shown. It can be observed that corn cob residues contain 0.4%, while BCM shows a significant increase with 4.0%, which is lower compared to what was reported by Zhao et al. (2018); these authors determined 10.79% of ash content when heating at 300 °C and 23.27% at 500 °C [30]. 

The results shown in Table 2 are also different from those reported by Mohan et al. (2014) and Xie et al. (2021), who used corn as biomass and the same carbonization temperature [26,31]. For BCM_Fe_, an 8.0% ash content was observed, a higher amount compared to BCM, which was attributed to the presence of inorganic particles, such as iron oxides, on the surface of BCM_Fe_, due to the melting point of iron being >1500 °C.

The biochar yield varied according to the type of biomass and the temperature used in the pyrolysis process, according to Amalina et al. (2022) [32]. Table 3 shows a comparison with biochars reported in the literature. For BCM, the results are similar to those reported by Mohan et al. (2014) [31]. However, as reported by Suo et al. (2021), an increase in the percentage can be attributed to a lower carbonization temperature [33]. 

### 3.2. Inorganic Composition

In order to determine the ash composition, XRF analysis was performed. The results are shown in Table 4, where it can be observed the presence of elements such as K, P, and Si, which are in a higher proportion in the BCM biochar when compared with corn cobs. The amount of K increases considerably by the pyrolysis process, being the main element in BCM with a concentration greater than 11%, similar to that reported by Gómez-Vásquez et al. (2022) [36]. The high amount of elements was attributed to the fertilizer used for plant growth (Land and Water Division, FAO, 2002) since the main elemental composition is K, P, and Si with concentrations greater than 0.6% [37]. Other elements such as Cl, Fe, and S were also detected, albeit in lower concentrations. For BCM_Fe_, an increase in Fe amount (8.761%) was attributed to the formation of iron oxides by the coprecipitation method.

### 3.3. Morphological Analysis

**Figure 1:**
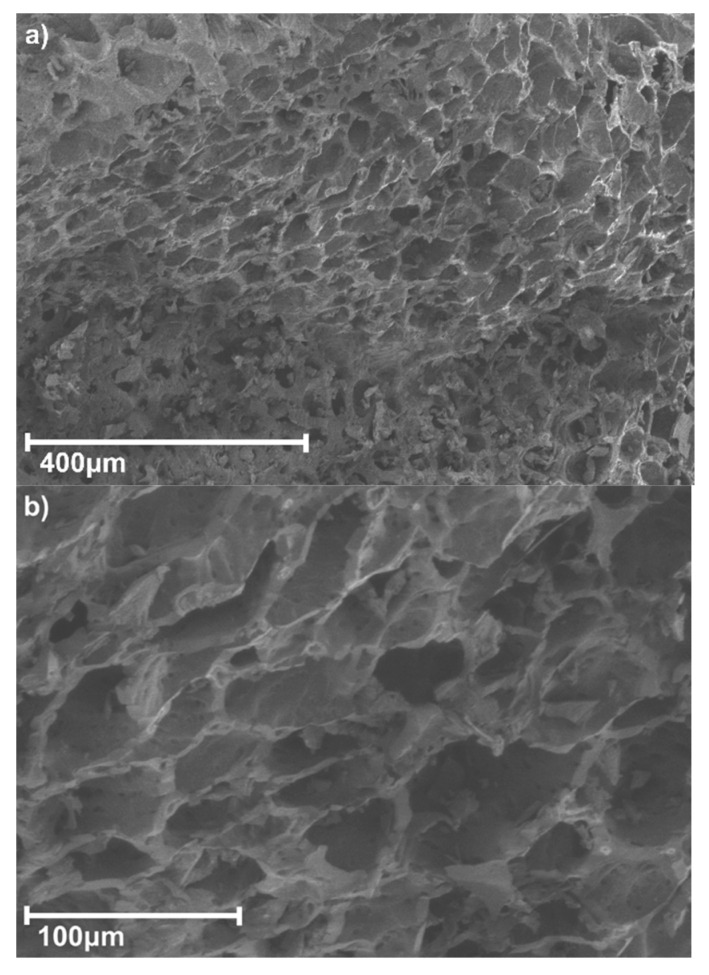
SEM images of the BCM structure, (**a**) 400 μm and (**b**) 100 μm.

SEM analysis was performed to characterize the surface morphologies of the samples. Figure 1 shows the BCM surface, where a highly porous structure, a large surface area (S_BET_: 1013.52 m^2^/g), and evenly distributed pores were observed. The pore size range was determined as mesopores and macropores, as micropores are generally more difficult to detect, according to the report by Quillope et al. (2021) [38].

**Figure 2:**
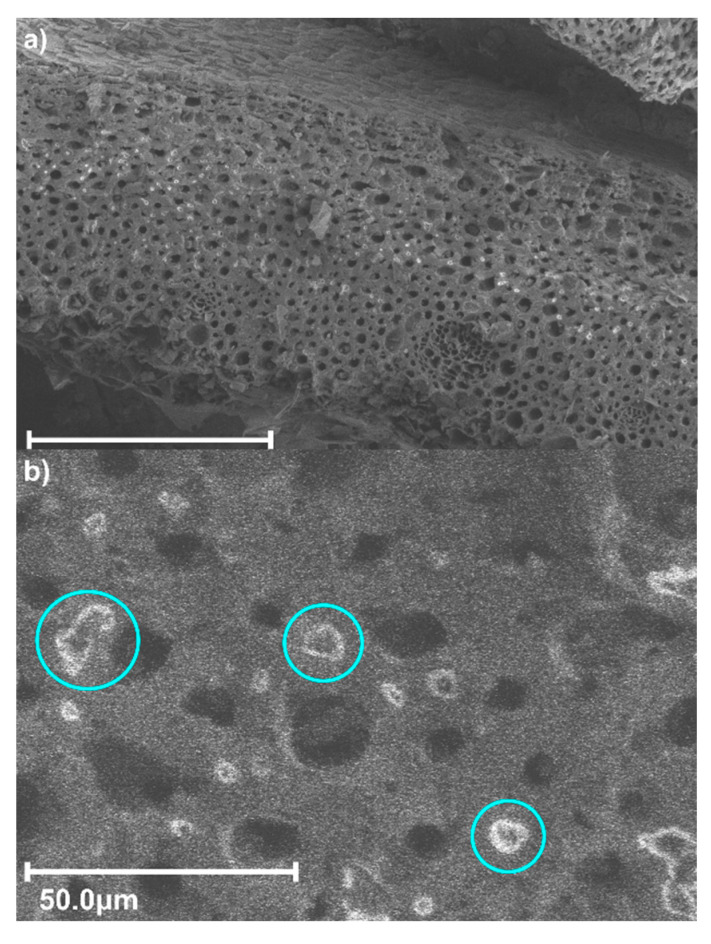
SEM images of the BCM_Fe_ structure, (**a**) 300 μm and (**b**) 50 μm. The blue circles represents the Fe particles observed in SEM.

The surface structure (S_BET_ = 903.67 m^2^/g) of magnetic biochar (BCM_Fe_) is shown in Figure 2. Particles with an intense brightness were observed, indicating the presence of an element with a higher atomic mass such as Fe [39]. Fe_3_O_4_ particles generally adopted a spherical shape and formed agglomerates, which were distributed over the biochar surface, according to the reports by Ouyang et al. (2017) and J. Yan et al. (2020) [40,41]. It can be determined from the SEM analysis of Figure 2 using Image-Pro Plus software that the average agglomerate diameter is 5.83 ± 2.23 μm. This is in accordance with the value reported by Navarathna et al. (2019), in which the agglomerated particle size was up to 7 μm [42].

**Figure 3:**
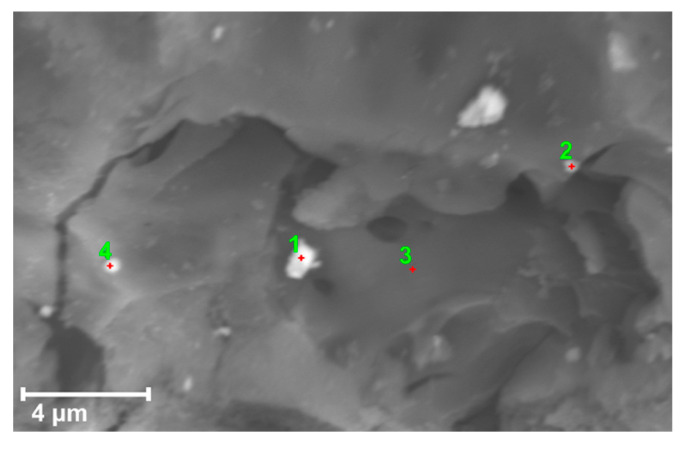
EDS image of the 4 BCM_Fe_ points analyzed. The numbers represents the point where EDS was done for Table 5.

The BCM_Fe_ surface elemental composition was obtained by EDS analysis. Four points of the sample (see Figure 3) were analyzed to obtain the mass percentage of each element (Table 5). Biochar BCM consists mainly of C (carbon) and O (oxygen). Other elements were also detected, although in smaller proportions. The results were consistent with those obtained by XRF analysis for Si, K, and Fe. No presence of Cl was observed in the sample, so Fe ions were transformed into iron oxide. In addition, the Fe percentage increased significantly due to the presence of Fe particles in the biochar. 

### 3.4. Structure of Modified Biochar

**Figure 4:**
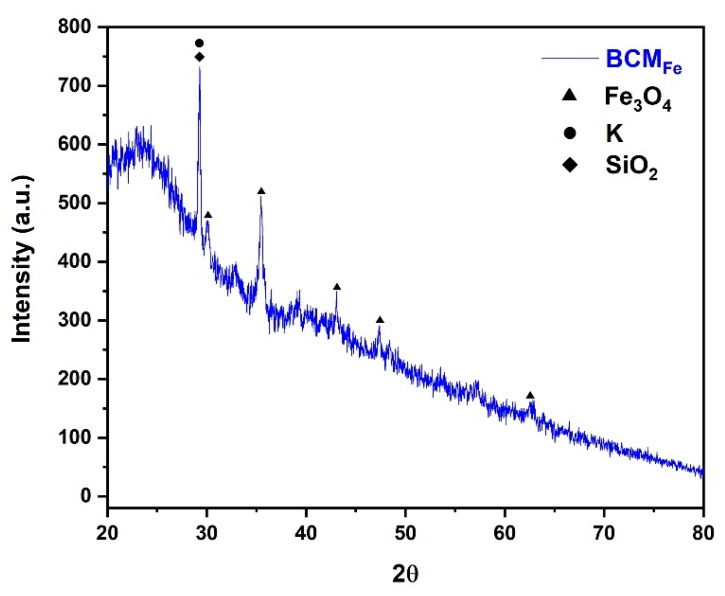
BCM_Fe_ XRD pattern.

The crystalline structures of the modified biochar were characterized by X-ray diffraction. The XRD pattern of the BCM_Fe_ is presented in Figure 4, which confirms that the precipitated iron ions are mainly magnetite (Fe_3_O_4_). A series of diffraction peaks corresponding to Fe_3_O_4_ were detected at 2θ values of 30.02°, 35.45°, 43.08°, 47.38°, and 62.53°, with Miller indices of (220), (311), (400), (331), and (440), respectively. The observed signals coincided with the diffraction patterns of Fe_3_O_4_ reported by Compeán-Jasso et al. (2008) and Peña-Rodríguez et al. (2018), confirming the successful formation of magnetite on the biochar surface [43,44]. 

In Figure 4, an intense signal at 29.29° due to silicon oxide (SiO_2_) with crystallographic chart 96-900-0809 can be observed. Since SiO_2_ is a compound that is prominent in the inorganic components of corn cob [36], or it may belong to a potassium mineral (K) with crystallographic chart 96-901-1978, these elements can be found in biochar due to the inorganic elements present in the biomass that was not carbonized. The signals for amorphous graphite were also observed at signals between 20 and 30° and 40 and 50°.

**Figure 5:**
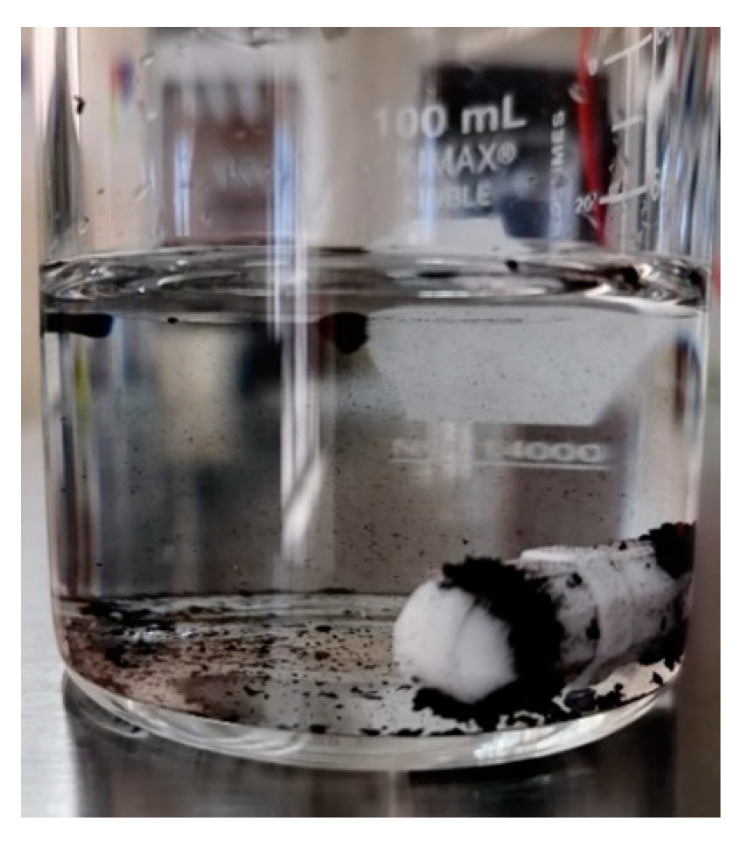
Magnetic properties of biochar (BCM_Fe_).

In order to identify if the material had the characteristic magnetic properties of magnetite, a simple test was carried out with the addition of a magnet to a suspension with 0.1 g of the magnetic biochar, and as can be seen in Figure 5, the material stuck to the magnet due to the magnetic properties. 

### 3.5. Surface Functional Groups

**Figure 6:**
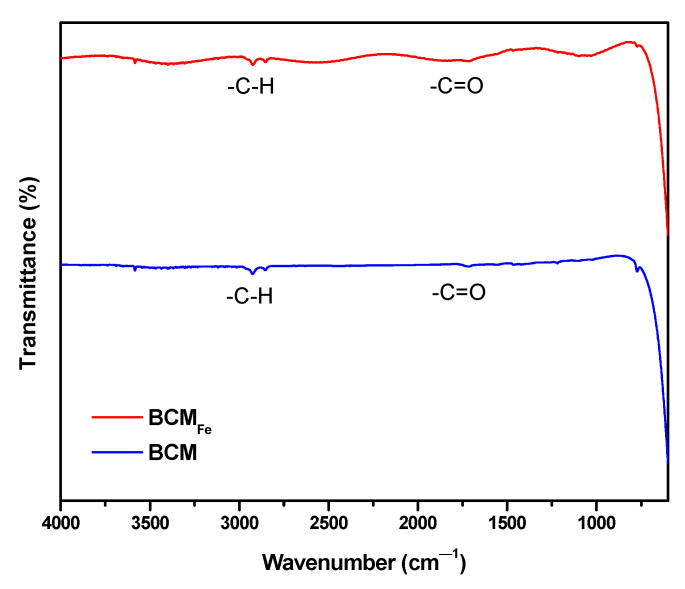
FTIR analysis of BCM and BCM_Fe_.

An FTIR analysis was performed to observe surface functional groups on the obtained biochars. The results are presented in Figure 6, where the low intensity of the signals, which are attributed to the temperature at which the pyrolysis process was carried out, can be observed. According to Manoharan et al. (2022), the number of functional groups decreases at higher temperatures due to the release of CO_2_ [45]. 

Both biochars showed similar bands, one observed at 1712 cm^−1^, corresponding to the C=O stretching vibration of the carbonyl group [46], which is formed during the carbonization process, and the double signals between 2800 and 3000 cm^−1^, corresponding to -C–H aliphatic compounds stretching vibrations [35,47]. The band near 500 cm^−1^ could be described as a stretching vibration of metallic oxides due to the presence of inorganic compounds in BCM and BCM_Fe_ [48].

### 3.6. Thermal Behavior

**Figure 7:**
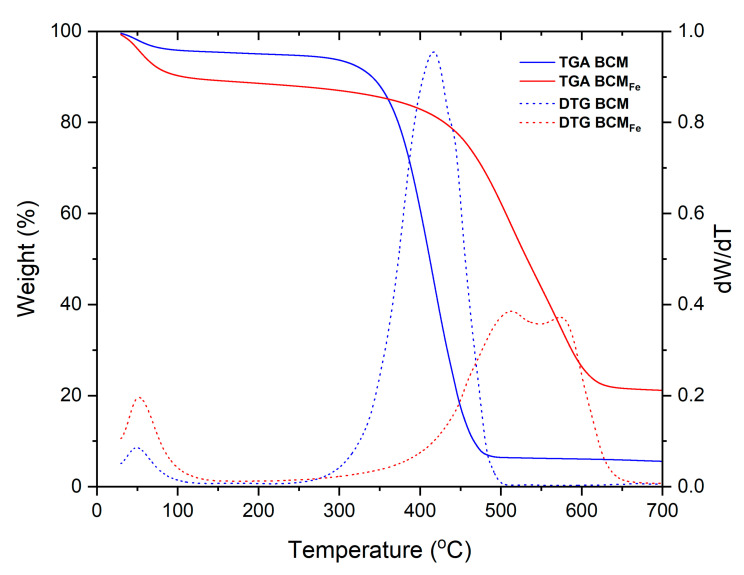
BCM and BCM_Fe_ biochar thermograms.

Thermal analysis was carried out on both biochars (BCM and BCM_Fe_). The thermogravimetric (TG) and differential thermogravimetric (DTG) curves of Figure 7 show two main weight loss processes for both biochars. In the first curve, BCM showed a 4% loss at 89 °C, while BCM_Fe_ showed a 10% weight loss, due to water molecules evaporating from the biochar surface. At higher temperatures, a greater reduction in weight loss was observed. In BCM, a weight loss of 93.8% was observed at 500 °C, while a weight loss of 78.6% was observed in BCM_Fe_ at 650 °C. The loss was attributed to the decomposition of lignocellulosic biomass and extracts such as proteins, fats, and sugars, among others [14,49], results observed in FTIR analysis, in addition to the loss of functional groups such as carboxyl and hydroxyl groups [50]. Compared to BCM biochar, BCM_Fe_ carbonized at a higher temperature and showed higher thermal stability, which corresponded to a greater amount of inorganic components [51]. 

The difference between the amount of weight loss for BCM_Fe_ and BCM was 15.2% and corresponds to the F_3_O_4_ present on the biochar surface according to XRD results. The DTG curve of the impregnated biochar showed two signals between 400 and 650 °C, attributed to the Fe_3_O_4_ oxidation to α-Fe_2_O_3_ and γ-Fe_2_O_3_.

### 3.7. Methylene Blue Adsorption Capacity

**Figure 8:**
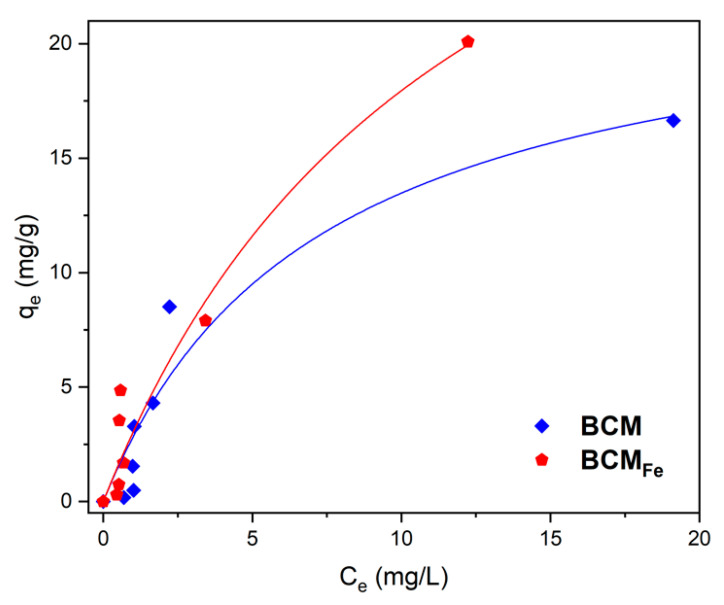
Methylene blue adsorption isotherm adjusted to the Langmuir model for the synthesized samples. pH = 7; T = 25 °C; dosage = 0.02 g/10 mL; methylene blue C_o_ = 1–25 ppm.

MB adsorption capacity was studied at pH 7 since the PZC of BCM was measured as 7.06. The adsorption capacity was adjusted to the Langmuir model, as can be seen in Figure 8. Non-linear regression was used to adjust the experimental data using Origin^®^ software. BCM biochar showed a maximum adsorption capacity (q_m_) of 23.17 mg/g and BCM_Fe_ biochar of 39.66 mg/g, as shown in Table 6. 

The q_m_ of BCM_Fe_ biochar is similar to that reported by Zeng et al. (2021), with a q_m_ of 39.35 mg/g at a temperature of 35 °C, using a sewage sludge-based magnetic biochar (Fe_3_O_4_) by pyrolysis for the removal of methylene blue in water [22]. Mubarak et al. (2015) prepared a magnetic biochar derived from an empty fruit bunch treated with ferric chloride (FeCl_3_) to remove methylene blue, obtaining a q_m_ of 31.25 mg/g [52], while Ruthiraan et al. (2017) obtained a qm of 46.30 mg/g in MB removal with a magnetic iron oxide biochar (impregnated with Fe_2_O_3_ solution) obtained from mangosteen peel [53].

In addition to q_m_, Table 6 shows the k_L_, R_L_, and R^2^ parameters. The Langmuir constant (k_L_) indicates the degree of interaction between the adsorbate and the surface. When the adsorption energy constant (k_L_) was less than 1, it indicates that there was a strong interaction between the adsorbate and the adsorbent. The Langmuir separation factor (R_L_) showed values lower than 1 in both biochars, which indicated an appropriate adsorption process. If R_L_ > 1 is not suitable, while if 0 < R_L_ < 1 is appropriate [54].

Further, the linear correlation coefficient (R^2^) was high, so the adsorption isotherms fit well for the Langmuir model. This model indicates that the adsorbent surface was completely flat and homogeneous and that each surface site can only hold one molecule of the adsorbate, resulting in monolayer-type adsorption [55,56]. The metal–π interaction is one of the mechanisms reported in the literature for organic compounds in modified biochars with metallic oxides [57,58,59,60]. By this, an increase in the MB adsorption capacity for BCM_Fe_ was observed, due to the presence of Fe_3_O_4_ particles on the surface. Several reports have corroborated that MB adsorption by activated carbons from lignocellulosic materials demonstrates a strong correlation with the Langmuir isotherm equation [61].

**Figure 9:**
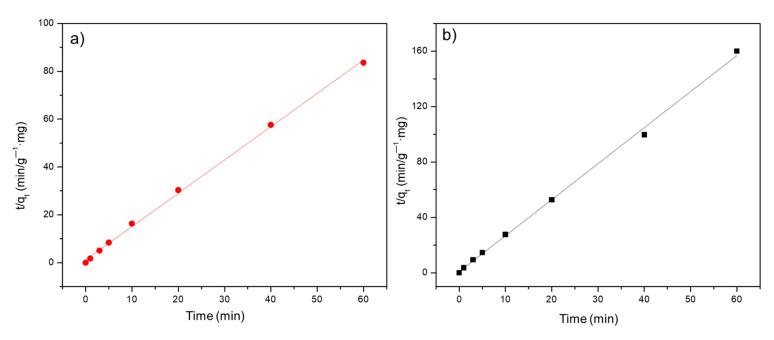
Kinetic PSO model adjust for BCM (**a**) and BCM_Fe_ (**b**).

Furthermore, experimental data were adjusted to the PFO and PSO order kinetic models; however, both biochars were better fitted to the PSO (R^2^ > 0.998) (Figure 9). The PSO model describes the adsorption process in a surface monolayer. The results agreed with those observed in adsorption isotherms. 

## 4. Conclusions

The results showed that the yield of BCM was 28.483%, higher than that of BCM_Fe_ (i.e., 26.160%). Both biochars show a highly porous surface, a large surface area, and uniformly distributed pores and they are mainly composed of C and O, in addition to Fe, Si, and K. BCM_Fe_ biochar contains a higher amount of Fe compared to BCM due to its impregnation with Fe ions, successfully achieving the addition of Fe_3_O_4_ particles, and XRD analysis supports this result. 

While the ash content of BCM was 4% lower than BCM_Fe_ (i.e., 8%), there were 4% more inorganic compounds in BCM_Fe_. Both biochars presented carbonyl and aliphatic groups on the surface. The TGA revealed that BCM carbonizes at 500 °C with a weight loss of 93.8%, while BCMFe carbonizes at 650 °C with a weight loss of 78.6%. The above indicates that BCM_Fe_ showed higher thermal stability. 

It was possible to obtain a material capable of removing the organic pollutant methylene blue, with a maximum adsorption capacity of 23.17 mg/g and 39.66 mg/g for BCM and BCM_Fe_ biochar, respectively. The modified biochar (BCM_Fe_) showed a higher adsorption capacity (i.e., 71.17%) than BCM. 

## Figures and Tables

**Table 1:** Experimental pyrolysis conditions and performance of BCM.

Sample Condition	N_2_ Flow Rate (mL/min)	Heating Rate (°C/min)	Yield (%)	Average Yield (%)	Error (%)
Raw	50	10	4.90	5.60	0.117
Raw	50	10	6.20		
Raw	50	10	5.70		
Raw	35	10	25.30	28.48	0.098
Raw	35	10	29.61		
Raw	35	10	30.54		
Cooked	35	5	29.24	26.91	0.081
Cooked	35	5	26.59		
Cooked	35	5	24.91		
Cooked	35	10	22.27	22.45	0.218
Cooked	35	10	27.43		
Cooked	35	10	17.64		

**Table 2:** Ash content of corn cob and BCM and BCM_Fe_ biochars.

Material	Ash Content (%)
Corn cob	0.4 (±0.04)
BCM	4.0 (±0.01)
BCM_Fe_	8.0 (±0.02)

**Table 3:** Comparison of yield and ash percentage for different biochars.

Biomass	Temperature (°C)	Yield (%)	Ash (%)	Reference
Water hyacinth	350	34.25	10.42	[34]
Corn straw	400	31.60	NR	[33]
Pigeon pea stalk	400	29.80	3.08	[35]
Corn stalk pellet	500	33.41	20.86	[26]
Corn stover	500	28.21	6.60	[31]
Sugarcane bagasse	550	21.15	1.16	[34]
Bamboo	600	27.00	4.65	[35]

Note: Not reported.

**Table 4:** Samples composition obtained by XRF.

Element	Corn Cob		BCM		BCM_Fe_	
	Concentration (%)	Error (%)	Concentration (%)	Error (%)	Concentration (%)	Error (%)
K	1.548	0.003	11.269	0.140	9.718	0.098
P	0.138	0.011	0.662	0.075	0.912	0.005
Si	0.786	0.033	0.604	0.309	0.478	0.361
Cl	0.174	0.003	0.555	0.414	1.236	0.047
Fe	0.529	0.002	0.208	0.027	8.761	0.152

**Table 5:** Elemental composition of BCM_Fe._

Element	% by Mass			
	Point 1	Point 2	Point 3	Point 4
C	28.84	44.33	55.91	55.78
O	8.08	27.75	22.67	31.85
Fe	46.06	7.50	3.76	4.78
Si	14.58	15.53	12.17	6.78
K	2.44	4.89	5.49	1.80
Total	100	100	100	100

**Table 6:** Langmuir model parameters.

Material	q_m_ (mg/g)	k_L_ (L/mg)	R_L_	R^2^ (%)
BCM	23.17	0.14	0.22–0.88	99.44%
BCM_Fe_	39.66	0.08	0.33–0.92	99.61%

## Data Availability

Not applicable.
